# Dramatic Variability of the Carbonate System at a Temperate Coastal Ocean Site (Beaufort, North Carolina, USA) Is Regulated by Physical and Biogeochemical Processes on Multiple Timescales

**DOI:** 10.1371/journal.pone.0085117

**Published:** 2013-12-17

**Authors:** Zackary I. Johnson, Benjamin J. Wheeler, Sara K. Blinebry, Christina M. Carlson, Christopher S. Ward, Dana E. Hunt

**Affiliations:** Marine Laboratory, Nicholas School of the Environment, Duke University, Beaufort, North Carolina, United States of America; University of Hong Kong, Hong Kong

## Abstract

Increasing atmospheric carbon dioxide (CO_2_) from anthropogenic sources is acidifying marine environments resulting in potentially dramatic consequences for the physical, chemical and biological functioning of these ecosystems. If current trends continue, mean ocean pH is expected to decrease by ~0.2 units over the next ~50 years. Yet, there is also substantial temporal variability in pH and other carbon system parameters in the ocean resulting in regions that already experience change that exceeds long-term projected trends in pH. This points to short-term dynamics as an important layer of complexity on top of long-term trends. Thus, in order to predict future climate change impacts, there is a critical need to characterize the natural range and dynamics of the marine carbonate system and the mechanisms responsible for observed variability. Here, we present pH and dissolved inorganic carbon (DIC) at time intervals spanning 1 hour to >1 year from a dynamic, coastal, temperate marine system (Beaufort Inlet, Beaufort NC USA) to characterize the carbonate system at multiple time scales. Daily and seasonal variation of the carbonate system is largely driven by temperature, alkalinity and the balance between primary production and respiration, but high frequency change (hours to days) is further influenced by water mass movement (e.g. tides) and stochastic events (e.g. storms). Both annual (~0.3 units) and diurnal (~0.1 units) variability in coastal ocean acidity are similar in magnitude to 50 year projections of ocean acidity associated with increasing atmospheric CO_2_. The environmental variables driving these changes highlight the importance of characterizing the complete carbonate system rather than just pH. Short-term dynamics of ocean carbon parameters may already exert significant pressure on some coastal marine ecosystems with implications for ecology, biogeochemistry and evolution and this shorter term variability layers additive effects and complexity, including extreme values, on top of long-term trends in ocean acidification.

## Introduction

Carbon dioxide concentrations are rising at ~3% per year in both the atmosphere and oceans. The rise of *p*_*CO*2_ in the oceans leads to increases in dissolved inorganic carbon (DIC) and decreases in pH to maintain equilibrium among the major components of the carbonate system including carbon dioxide (CO_2_), carbonic acid (H_2_CO_3_), bicarbonate (HCO_3_^-^) and carbonate (HCO_3_^2-^). In the open ocean, this increase in DIC has resulted in an increase in ocean acidity with a mean pH decrease of 0.002 units per year over the last ~20 years [1,2]. Unmitigated, increasing *p*_*CO*2_ -driven acidification is expected to continue for the foreseeable future resulting in a mean ocean pH decrease of ~0.2 units over the next 50 years [3]. This changing ocean pH and carbonate chemistry will affect a broad spectrum of physical and biogeochemical properties of the ocean ecosystems including transmission of sound, metals chemistry as well as the physiology, growth and reproduction of numerous organisms, especially those that deposit carbonate (e.g. corals, coccolithophorids and molluscs) and others [4-7]. Thus, identification and quantification of explanatory variables that drive observed patterns in the marine carbonate system is critical to understanding the effects of globally increasing *p*_*CO*2_ on ocean ecosystems.

Layered on top of long-term acidification is spatial and temporal variability due to a variety of physical and biogeochemical drivers. For example, due to their increased sensitivity to rising *p*_*CO*2_ some oceanographic regions such as the Eastern North Pacific already experience ocean acidification beyond this 0.2 pH unit projected average global ocean decrease [8-11]. Other locations, notably including coastal areas, experience dramatic changes in some carbon system variables over short time frames and spatial scales [12-14], demonstrating the highly dynamic nature of the coastal marine carbon system [15-18]. Thus, the coastal ocean has recently been highlighted as an area where there is a critical need to quantify the natural range and variability of the total carbonate system as well as the mechanisms leading to these changes. Increased coastal monitoring is required because of larger potential variability and the increased risk of human impacts and interactions with these systems [19]. 

Here we describe results from a coastal, mesotrophic ocean site located at the Beaufort Inlet (Beaufort, NC USA) where water from the Newport River Estuary mixes with waters from the coastal Atlantic Ocean. Thus this site integrates both marine and terrestrial influences on coastal ocean carbonate parameters. The Beaufort Inlet is part of the second largest estuarine system on the US East Coast (Neuse-Pamlico) and situated in the middle of the temperate latitudes, where most humans live; characterizing natural variability at this location provides a benchmark for longer-term studies of ocean acidification and its potential impacts on an ecosystem of critical importance for humans. This time-series, which samples over multiple time scales, shows that changes in pH on sub-year time scales exceed the magnitude of long-term projections and are driven by a combination of environmental drivers including temperature, alkalinity, primary production/respiration, water mass movement (e.g. tides) and stochastic events (e.g. storms). These results have implications for interpreting the effects of longer term trends in global ocean acidification.

## Materials and Methods

### Sample collection

All samples were taken as part of the Pivers Island Coastal Observatory (PICO) time-series, Beaufort, NC, USA at 34.7181 °N 76.6707 °W at a location with a mean low tide water column depth of ~4.5 m. Water was sampled using a 5 L niskin bottle centered at 1 m with a bottle length of 0.7 m. DIC was measured in triplicate on mercuric chloride poisoned samples by acidification and subsequent quantification of released CO_2_ using a CO_2_ detector (Li-Cor 7000) [20]. pH was measured spectrophotometrically with m-cresol purple [21] in triplicate at standard temperature (25°C) and reported on the log total hydrogen ion scale (pH_*T*,25°C_). Both DIC and pH samples were collected following recommended procedures [20] and measurements were calibrated against Certified Reference Materials purchased from Dr. A. G. Dickson of the Scripps Institution of Oceanography, University of California, San Diego. Nutrients (NO_2_, NO_3_, PO_4_, SiOH_4_) were measured in duplicate on 0.22 µm filtered samples stored at -80°C until later analysis using an Astoria-Pacific A2 autoanalyzer following the manufacturer’s recommended protocols. Salinity was measured using a calibrated handheld conductivity, temperature and depth instrument (YSI Castaway) or on discrete samples using a refractometer calibrated against standards. Duplicate chlorophyll pigment samples were extracted in MeOH and measured fluorometrically as previously described [22]. Temperature was measured in duplicate using NIST traceable thermocouples. Oxygen was measured optically *in situ* using a calibrated probe (YSI ProODO). Secchi depth was measured in duplicate using a 20 cm disk with four alternating white and black quadrants. Turbidity was measured in duplicate on discrete samples using a calibrated handheld turbidimeter (Orion AQ4500). Tidal height was obtained from US NOAA for station #8656483 (Beaufort, NC). Day length and incoming no-sky solar radiation were estimated using the USGS MATLAB AIR_SEA Toolbox <http://woodshole.er.usgs.gov/operations/sea-mat/air_sea-html/index.html>. Hourly precipitation data for Morehead City (COOP:315830) over the period 1/1/2001 - 1/1/2012 was obtained from the National Oceanic and Atmospheric Administration's National Climatic Data Center. No specific permissions are required for these activities at this sampling site and field studies did not involve endangered or protected species. All data generated for this study are freely publically available through BCO-DMO <http://bco-dmo.org>

### Data handling

Total alkalinity (TAlk), *p*_*CO*2_and pH_T_ (i.e. pH_T_ at the *in situ* temperature) were calculated from measured pH_*T*,25°C_ and DIC and other state variables using CO2SYS [23]. A major rainfall event was defined as a storm resulting in ≥50.8 mm precipitation (≥2σ) over a 24 h period. Principle component analysis (PCA) and hierarchical clustering analysis (HCA) were performed on the weekly dataset with the full set of environmental variables [barometric pressure, dissolved oxygen, oxygen saturation, projected daily insolation, mean lower low water tidal height (MLLW), chlorophyll a, salinity, Secchi depth, temperature, dissolved inorganic carbon (DIC), nitrite (NO_2_), nitrate (NO_3_), silicate (SiOH_4_), phosphate (PO_4_), pH_T_]. Prior to analyses, values were scaled to unit variance for each variable in the dataset. The scaled data matrix was subjected to PCA via singular value decomposition. The first two principal components accounted for 59.02% of the variance (PC1: 33.97%; PC2: 25.05%). Hierarchical clustering analysis was performed using the complete linkage method on a distance matrix calculated from the scaled dataset. PCA and HCA were implemented using the R Stats Package 2.13.1 for R.

## Results and Discussion

**Figure 1:**
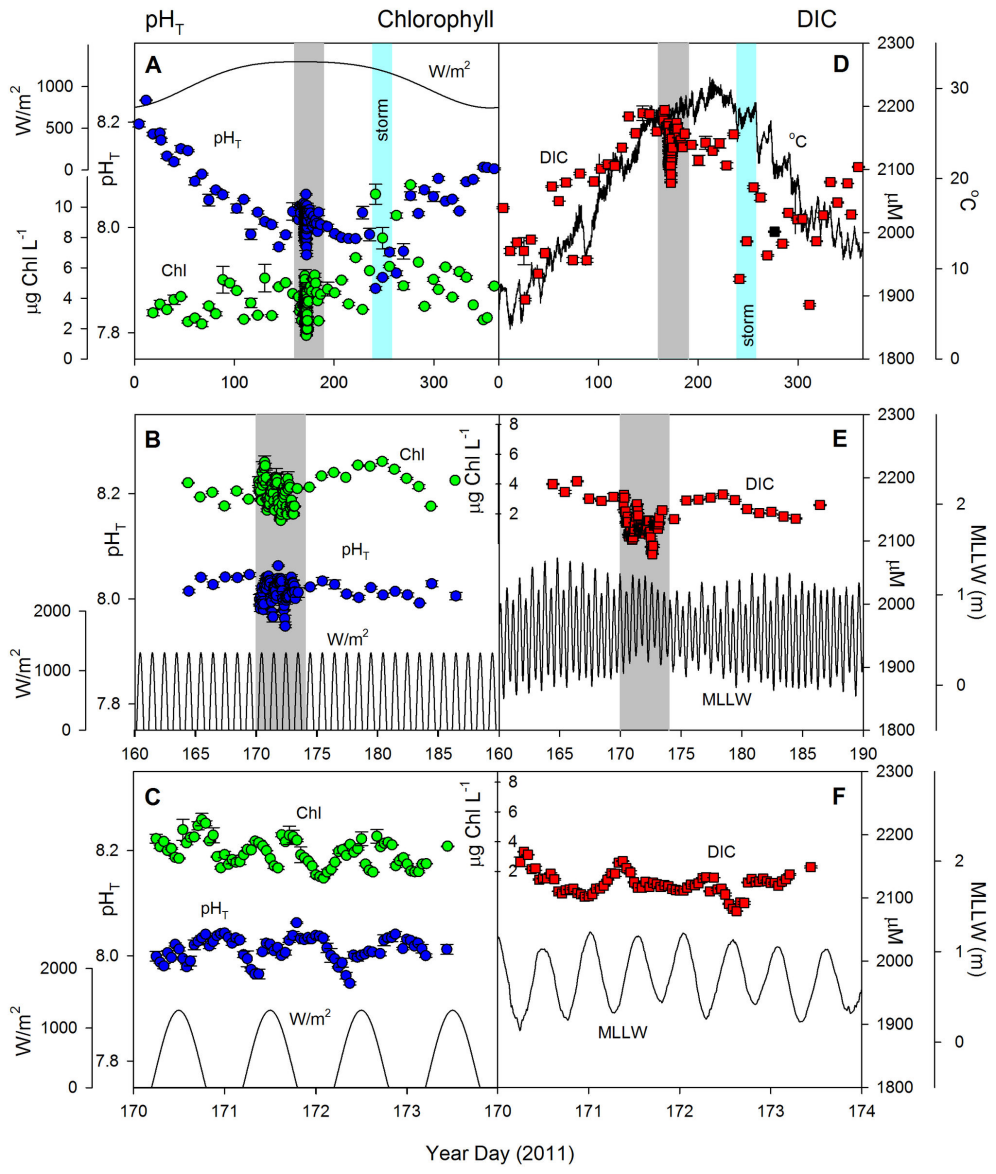
Nested temporal variability of carbon and environmental parameters at the Pivers Island Coastal Observatory study site. Plots depict pH_T_ (*in* *situ*: A-C), chlorophyll (µg L^-1^: A-C), DIC (µM: D-F) and associated physical variables including incoming no-sky solar radiation (W m^-2^: A-C), water temperature (°C: D), or tidal height (MLLW, m: E-F). Data are shown depicting the nested sampling design with weekly measurements over the course of the year (A, D), daily measurements over a 3 week period (B, E) and hourly measurements over 3 days (C, F) Gray bars indicate periods of more intense sampling, shown in the panel immediately below. Cyan bars indicate periods influenced by major storm events. For clarity, only the maximum daily W m^-2^ is plotted in top row.

We focus on quantifying pH and associated environmental variables of a dynamic coastal estuary as part of the Pivers Island Coastal Observatory (PICO), Beaufort, NC as a representative temperate coastal environment important for recreationally and commercially important juvenile and adult marine biota and like many coastal locations, it is a site of high biological activity [24]. The Beaufort Inlet is part of the second largest estuarine system on the US East Coast (Neuse-Pamlico), and thus characterizing natural variability at this location provides a key benchmark for longer-term studies of ocean acidification. In addition, estuary systems in general and the US East Coast in particular are poorly characterized in terms of the temporal variability of their carbonate systems. Using a nested temporal sampling strategy, we measured two key variables of the marine carbon system (DIC and pH), and thus constrain the carbonate system allowing calculation of alkalinity and *p*_*CO*2_ along with measurements of many environmental variables that may explain or be altered by changes in pH and DIC. Similar to open ocean environments, pH_T_ (pH at *in situ* temperature, reported on total hydrogen ion scale) and DIC exhibit dramatic temporal variability in the coastal ocean over a variety of time scales [25] (Figure 1). Some of the most profound changes are the annual cycles in both pH_T_ and DIC, (a range of ~0.3 and 200 µM respectively) with a minimum in pH_T_ and a maximum in DIC in the summer (Figures 1A, 1D and S1). If the carbonate system was in equilibrium and the sole driver of changes in pH, elevated levels of DIC would directly lower pH_T_. However, based on time-lagged correlation the summer minimum in pH_T_ precedes the maximum in DIC by ~2 months pointing to different mechanisms driving their respective extremes (Figure S2). Over the annual cycle pH_T_ is strongly correlated with temperature (r^2^=0.68), whereas DIC is most strongly correlated with salinity (r^2^=0.69) (Figures 1 and S3). Increased temperature lowers pH_T_ due to reduced gas solubility and changes in carbonate chemical speciation [26] and this affects the carbonate system in open ocean ecosystems as well [27]. Similarly, dissolved oxygen is strongly correlated with pH_T_ (r^2^=0.71) and temperature (r^2^=0.82) with the highest dissolved oxygen observed in the winter, also due to temperature-driven changes in gas solubility (Figure S3). Further supporting the importance of temperature in regulating pH_T_, temperature normalized pH (pH_*T*,25°C;_) is maximal in the summer (the opposite of *in situ* measurements) (Figure S4). The seasonal cycle in DIC is also correlated with temperature (r^2^=0.52), but not water clarity (Secchi depth or chlorophyll, p>0.05) also supporting its importance (Figures 1 and S3). 

With measurements of both DIC and pH_T_ we calculate total alkalinity (TAlk), a measure of the buffering capacity of seawater. TAlk is also maximal in the summer, coincident with the peak in DIC (Figure S5), which is the major buffer in marine systems. Similar to patterns in the open ocean [28], TAlk is highly correlated with salinity (r^2^=0.78) and may be related to evaporation-driven concentration of ions in the shallow estuary or physical mechanisms of water mass transport, but does not appear to be driven by changes in PO_4_ or SiOH_4_ which are components of TAlk. Nevertheless, changes in salinity alone do not account for the magnitude of change in TAlk or DIC [23] suggesting additional biogeochemical processes influencing this variable that perhaps originate from benthic or terrestrial sources.

While seasonal-scale temporal variability is largely correlated with physical mechanisms, higher resolution daily measurements of pH_T_ and DIC exhibit little coherent variability with environmental variables (Figure 1B & E). However, hourly measurements of pH_T_ and DIC over a 72 h period in the summer both show strong diurnal patterns that are apparently driven by biological activity (Figure 1C & F). pH_T_ varies by ~0.1 unit over the day/night cycle and is closely tied to insolation, highlighting the importance of microbial-mediated autochthonous primary production and respiration in modulating short-term changes in pH_T_. This diurnal variability is 10-times greater than daily changes in open ocean ecosystems [1] and is the result of higher biomass and biological activity in coastal waters. The maximal rate of increase in pH_T_ is coincident with the peak in insolation and is consistent with photosynthetic uptake of CO_2_ and a subsequent increase in pH_T_ and decrease in DIC (Figure 1C & F). After sunset, pH_T_ begins to decrease and DIC increase as respiration exceeds CO_2_ uptake; diurnal variability in temperature (~2°) and salinity (~3 units) at this site alone do not explain the observed differences in pH_T_ and DIC. Thus these high frequency changes in DIC and pH_T_ are most strongly tied to biological activity rather than photosynthetic biomass (chlorophyll concentration) per se; on these timescales biomass is primarily regulated by the tidal cycle with high tide increasing the contribution of oligotrophic ocean waters with relatively lower chlorophyll concentrations to the study site (Figure 1C). DIC, which varies diurnally by ~50-75 µM, is anticorrelated with pH_T_, but similar to pH_T_ it shows a diel pattern driven by biogeochemical cycling of CO_2_ (Figure S5). During the day, drawdown of CO_2_ by photosynthetic primary production dominates, while in the dark respiration dominates, releasing CO_2_ into coastal waters (Figure S5) [29,30]. These diurnal changes in pH_T_ and DIC track oxygen saturation that has maxima and minima at the same time point as DIC and pH extremes (Figure S6). In the summer, the diel change in DIC can exceed 75 µM, with a secondary signal (~10 - 20 µM) due to tidal flows of lower DIC, oligotrophic ocean water.

**Figure 2:**
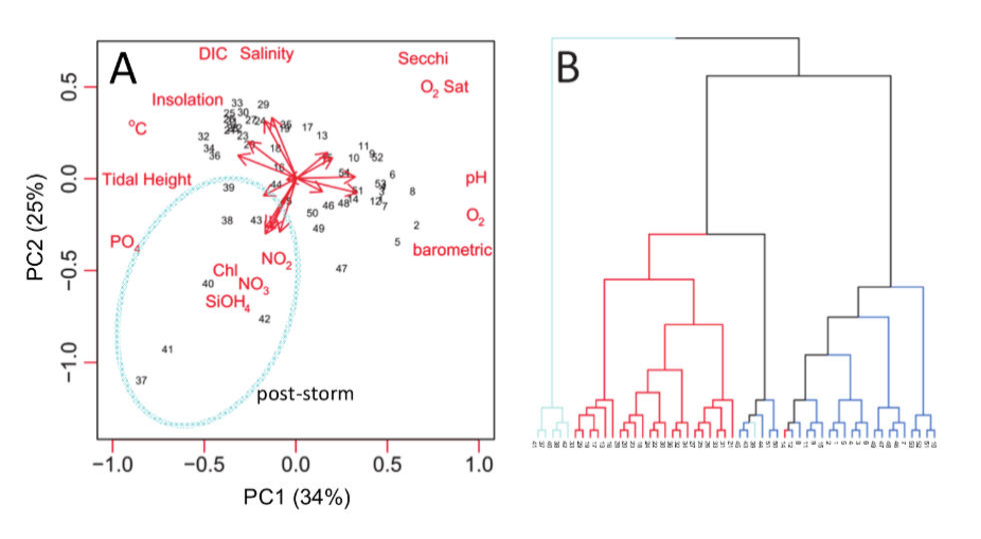
Non-dimensional analyses of weekly samples (2011) from the Pivers Island Coastal Observatory. A. Principle component analysis of the observed environmental variables. Cyan oval highlights environmental samples following significant storm events. B. Hierarchical clustering of time-series observations with color coded branches based on the season of sampling (blue=October-March, red= April-October, cyan= post-storm). In both panels sample numbers correspond to the week of sampling.

Superimposed on these periodic cycles in pH_T_ and DIC are episodic fluctuations that follow storms or other stochastic events that can dramatically influence coastal ocean biogeochemistry (and therefore the ecosystem’s carbonate system). For example, in the weeks following a large storm, pH_T_ can decrease by ~0.15 units (year day 229) and DIC increase by >200 µM (year day 236). This is similar to the magnitude of variability observed over an annual cycle (Figure 1), and much larger than the observed impacts of hurricanes on the carbonate system in the open ocean [31]. Further, following a major storm (Figure 1A & D), TAlk decreased by more than 300 µM indicating a large flux of acidic compounds, potentially from benthic, terrestrial or groundwater sources. These storm-driven fluctuations are linked to a substantial decrease in salinity (~4 units) as well as increases in turbidity (4.5 to 10.2 NTU), nutrient concentrations (NO_3_: <0.01 to 1.3 µM and SiOH_4_: 4.4 to 36.9 µM), and dramatic effects in other chemical and physical properties following a major rainfall. These dramatic effects were associated with a major storm event (Hurricane Irene, August 2011) that released up to 350 mm of rainfall over the watershed, which resulted in environmental conditions that were distinct from those at other times of the year (Figure 2). Together, these results show that storms that generate substantial terrestrial water inputs from both runoff and groundwater discharge play a key role in the carbon biogeochemistry of this region [32]. The frequency of these dramatic changes in the carbonate system is poorly constrained, but longer term decadal weather records of storm events suggest ~3 annual major rain events (≥2 SD above the mean, or ≥50.8 mm). The storm captured here illustrates how important periodic disturbances can be to the physical and biogeochemical environment of near-shore ecosystems; changes in pH arise due to direct physical and chemical modifications as well as due to indirect effects from increased biological activity following nutrient input into the system.

## Conclusions

Projected long-term trends in atmospheric and oceanic *p*_*CO*2_ will result in ocean acidification, which will in turn affect a range of marine processes and taxa [6,33]. In the oligotrophic open ocean, the larger spatial and temporal scales of physical processes [34] in conjunction with relatively lower biomass and biogeochemical activity including photosynthesis and respiration rates result in relatively small diurnal and annual variation in pH and DIC [1]. Yet in mesotrophic or eutrophic coastal oceans recent observations demonstrate that some ecosystems already experience annual or daily pH and DIC variability that vastly exceeds observed or predicted long-term changes in open ocean regions [15,16]. This dramatic hourly to annual variability in pH in the coastal ocean occurs over a range of temporal and spatial scales and encompasses physical and biogeochemical drivers including photosynthesis and respiration [35] and tidal mixing of water masses with different fluxes of CO_2_ (e.g. benthic, atmospheric, low biomass oligotrophic waters, high biomass mesotrophic waters). In addition to regular annual and daily cycles, episodic weather events increase the flux of fresh water into estuarine systems, and alter the carbonate system, resulting in the observed decrease in pH_T_ (and TAlk) following a storm (Figures 1 and 2). Overall, this study quantifies the variability of carbonate system components at the land-sea interface over multiple timescales; and this data suggests that coastal ecosystems and organisms already experience significant periodic (annual or diurnal) or episodic variability in pH and DIC. Indeed, observed annual variability (~0.3 units) and diurnal variability (~0.1 units) in coastal ocean acidity are both similar in magnitude to long-term global ocean projections (~0.2 units) associated with increasing atmospheric CO_2_. This observed short-term environmental variability may already exert significant ecological and evolutionary pressures on organisms [36] that are sensitive to pH or DIC (or TAlk and*p*_*CO*2_) [37,38] with cycling and episodic carbonate cycle extremes layered on top of long-term trends. Moreover, it is often extreme events rather than average conditions that determine the health of an ecosystem, thus environmental impacts of long-term ocean acidification may be first observed during short-term extreme events, such as the impact of high temperature excursions on coral bleaching [39] Additionally, this study points to additional key environmental variables such as photosynthesis and respiration rates or observational windows (e.g. post-storms) that will be critical for further understanding the mechanisms responsible for understanding ocean acidification at multiple time scales. Quantifying horizontal and vertical variability at appropriate scales will be important to understanding coastal ocean acidification mechanisms and processes. How these periodic and episodic changes impact the organisms of these ecosystems is not known.

**Figure 3:**
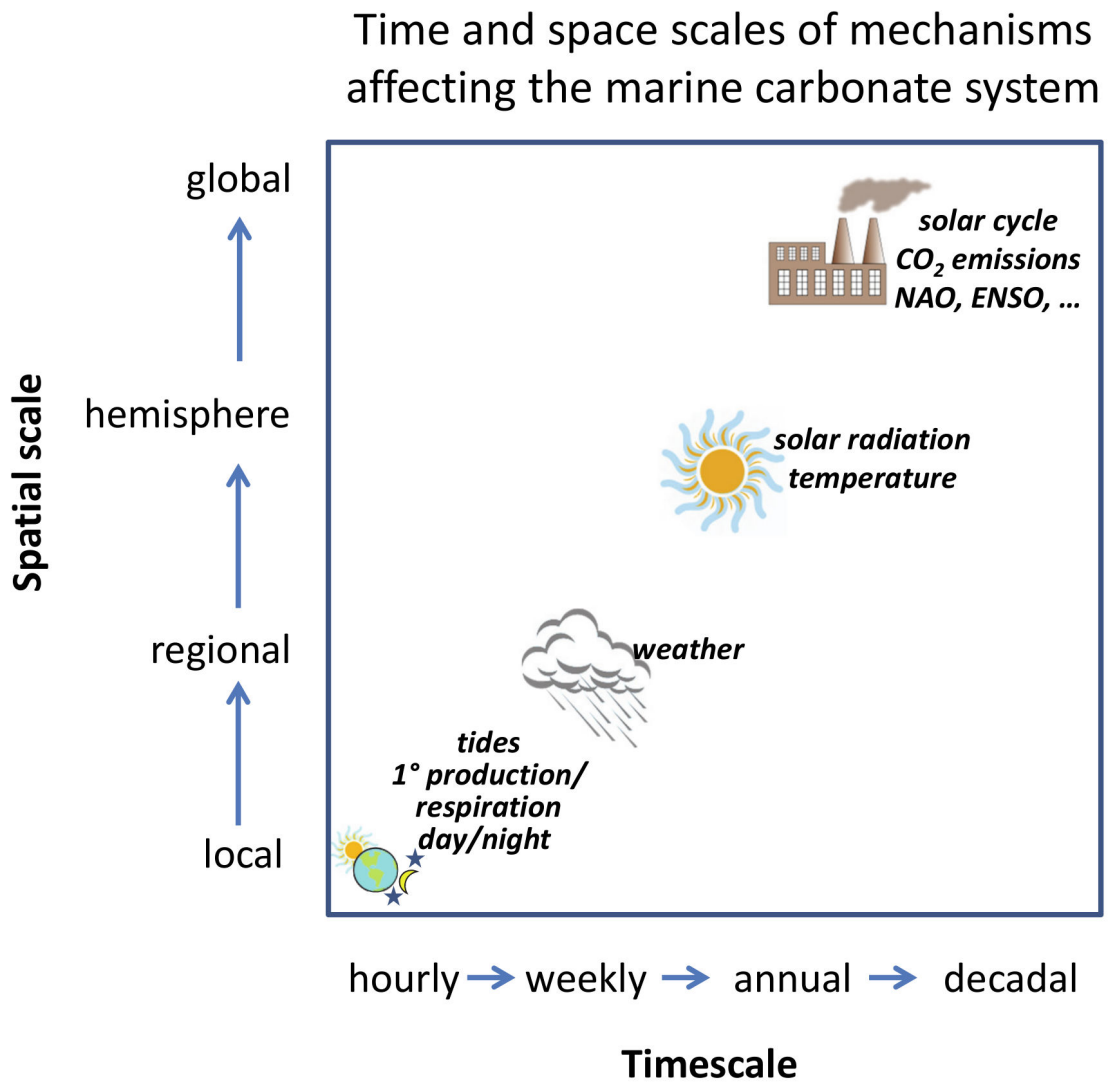
Conceptual diagram of major processes and space and time scales affecting the marine carbonate system in coastal marine ecosystems.

In order to predict the impacts of ocean acidification on marine systems, models should encompass both long-term trends and shorter term variability driven by physics and biogeochemistry at the appropriate spatial and temporal scales (Figure 3). More specifically, these models should account for the relatively greater dynamics in coastal versus open ocean ecosystems that are driven largely by enhanced biogeochemical rates and interactions at the sea-land interface [40]. Quantitatively characterizing this variability in the marine carbonate cycle and how it impacts the marine biota alone or in concert with other stressors [41,42] is essential to understanding the global impacts of ocean acidification in structuring coastal ecosystems and in making policy and management recommendations.

## Supporting Information

Figure S1 **Temporal variability of pH_T_ (*in situ*, blue) and DIC (µM, red) over a two year observation period at the Pivers Island Coastal Observatory site.**
(TIF)

Figure S2 **Cross-correlation between DIC (µM) and pH_T_ (*in situ*) over a two year observation period at the Pivers Island Coastal Observatory site.** Note the maximal (absolute value) correlation at ~60 days.
(TIF)

Figure S3 **Temporal variability of salinity (top panel, blue circles), oxygen (middle panel, green circles) and Secchi Depth (bottom panel, red circles) over a two year observation period at the Pivers Island Coastal Observatory site.** Error bars show 1 standard deviation.
(TIF)

Figure S4 **Temporal variability of pH_T_ (*insitu*, blue) and pH_T,25°C_ (black) over a two year observation period at the Pivers Island Coastal Observatory site.** Error bars show 1 standard deviation.
(TIF)

Figure S5 **Nested temporal variability of some carbon and environmental parameters at the Pivers Island Coastal Observatory study site for 2011.** Nested temporal variability of pCO_2_ (µatm: A-C), TAlk (µM: D-F) and associated physical variables including incoming no-sky solar radiation (W m^-2^: A-C), water temperature (°C: D), or tidal height (MLLW, m: E,F). Gray bars indicate periods of more intense sampling. Cyan bars indicate periods influenced by major storm events. Data are shown depicting the nested sampling design with weekly measurements over the course of the year (A, D), daily measurements over a 3 week period (B, E) and hourly measurements over a 3 day period (C, F) Gray bars indicate periods of more intense sampling, shown in the panel immediately below. For clarity, only the maximum daily W m^-2^ is plotted in top row.
(TIF)

Figure S6 **Nested temporal variability of percent oxygen saturation (filled circles) and incoming no-sky solar radiation (W m^-2^: A-C).** Gray bars indicate periods of more intense sampling. Cyan bars indicate periods influenced by major storm events. Data are shown depicting the nested sampling design with weekly measurements over the course of the year (A), daily measurements over a 3 week period (B) and hourly measurements over a 3 day period (C) Gray bars indicate periods of more intense sampling, shown in the panel immediately below. For clarity, only the maximum daily W m^-2^ is plotted in top row.
(TIF)
