# Supplementary figures and images for: Dramatic Variability of the Carbonate System at a Temperate Coastal Ocean Site (Beaufort, North Carolina, USA) Is Regulated by Physical and Biogeochemical Processes on Multiple Timescales
Source: PLoS One. 2013 Dec 17;8(12):e85117. doi: 10.1371/journal.pone.0085117 (PMC3866137; doi:10.1371/journal.pone.0085117)

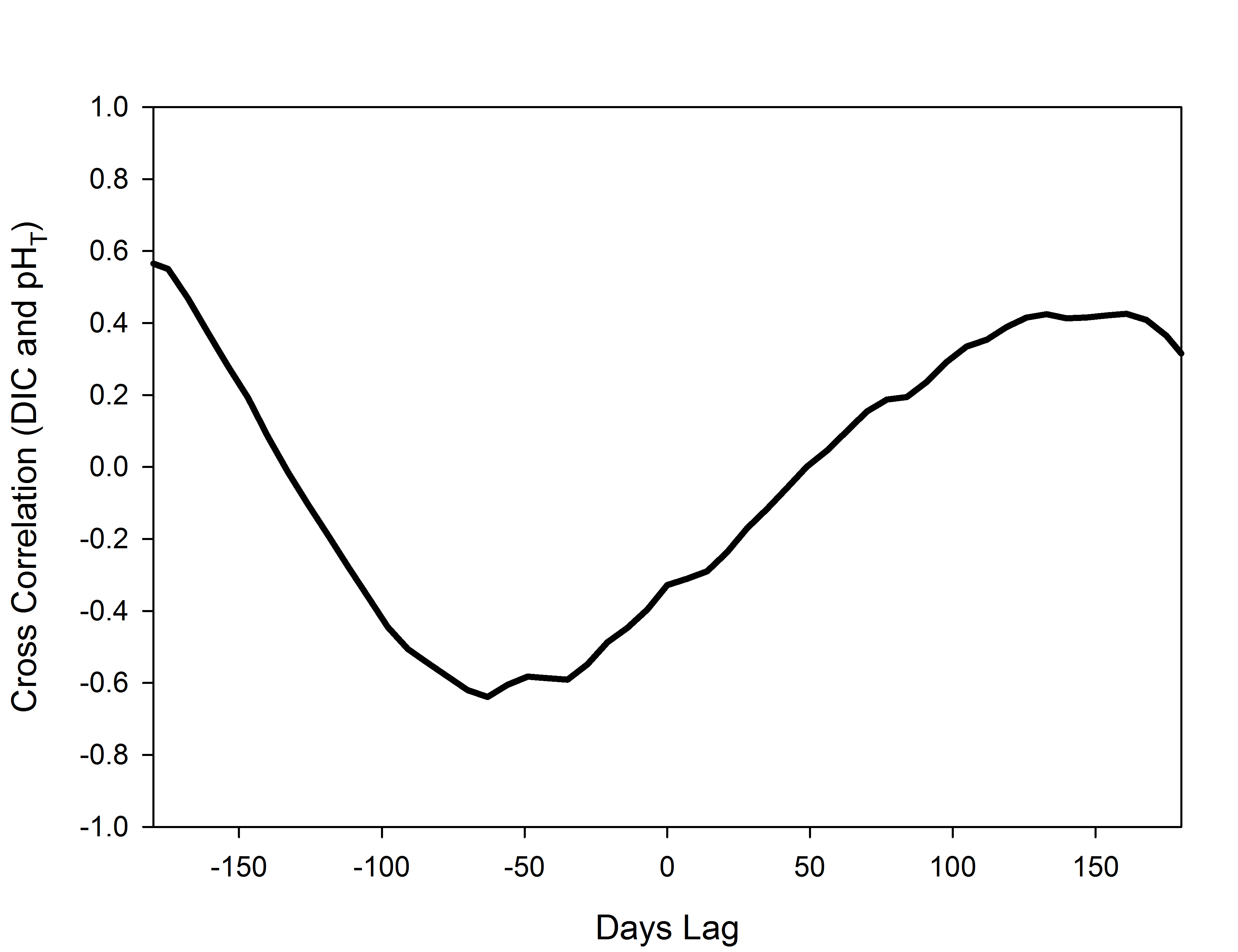

Supplement: Figure S2 — Cross-correlation between DIC (µM) and pHT (in situ) over a two year observation period at the Pivers Island Coastal Observatory site. Note the maximal (absolute value) correlation at ~60 days. (TIF) [file pone.0085117.s002.tif]

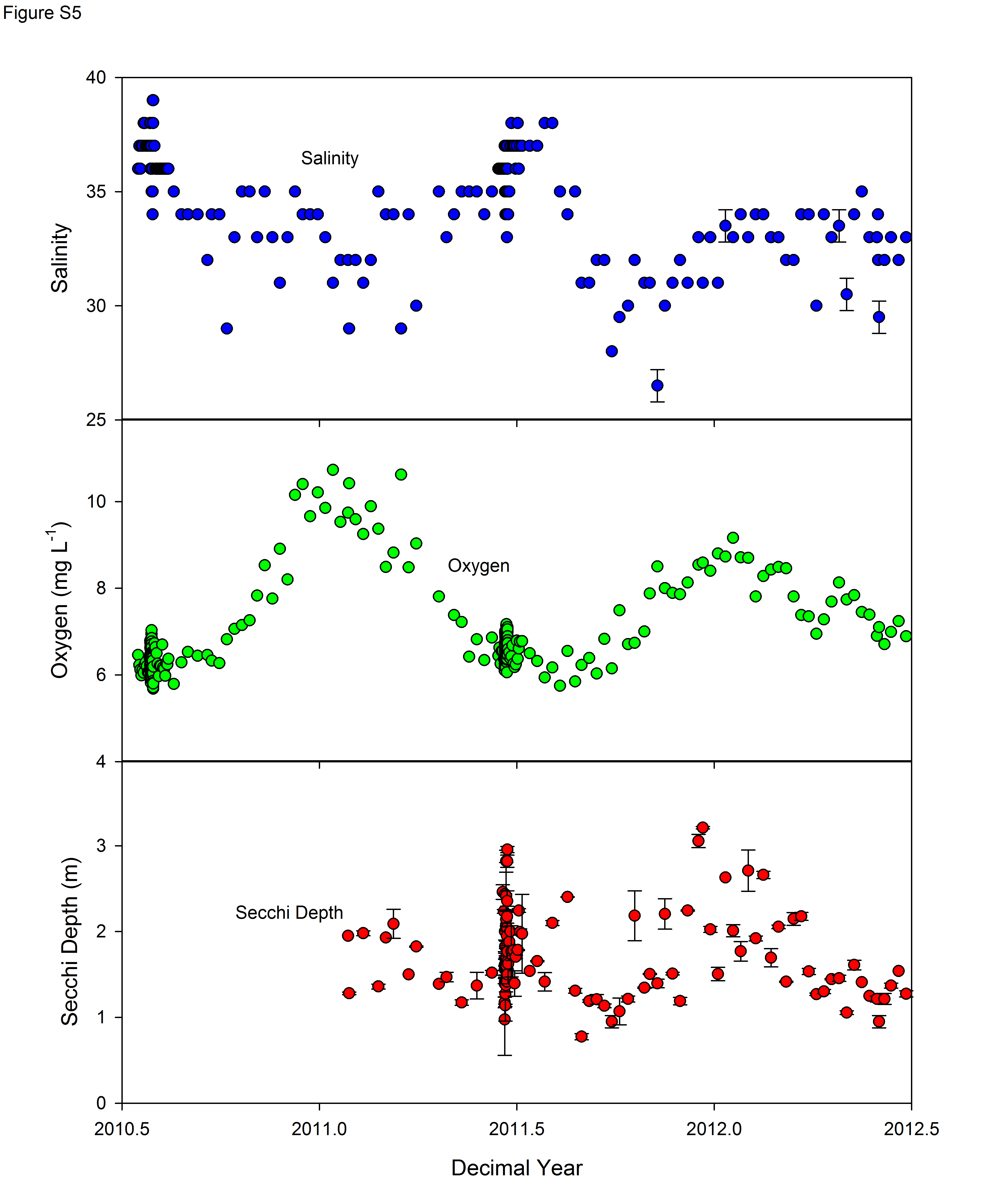

Supplement: Figure S3 — Temporal variability of salinity (top panel, blue circles), oxygen (middle panel, green circles) and Secchi Depth (bottom panel, red circles) over a two year observation period at the Pivers Island Coastal Observatory site. Error bars show 1 standard deviation. (TIF) [file pone.0085117.s003.tif]

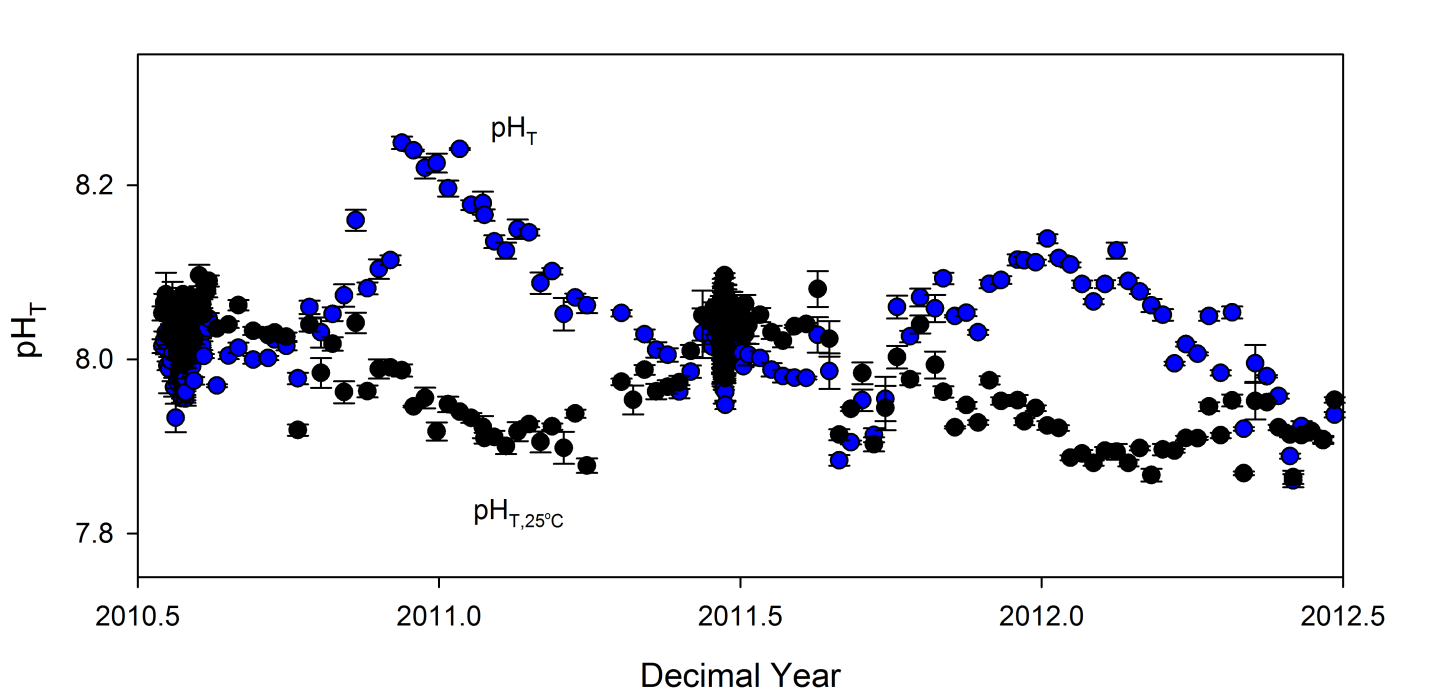

Supplement: Figure S4 — Temporal variability of pHT (insitu, blue) and pHT,25°C (black) over a two year observation period at the Pivers Island Coastal Observatory site. Error bars show 1 standard deviation. (TIF) [file pone.0085117.s004.tif]

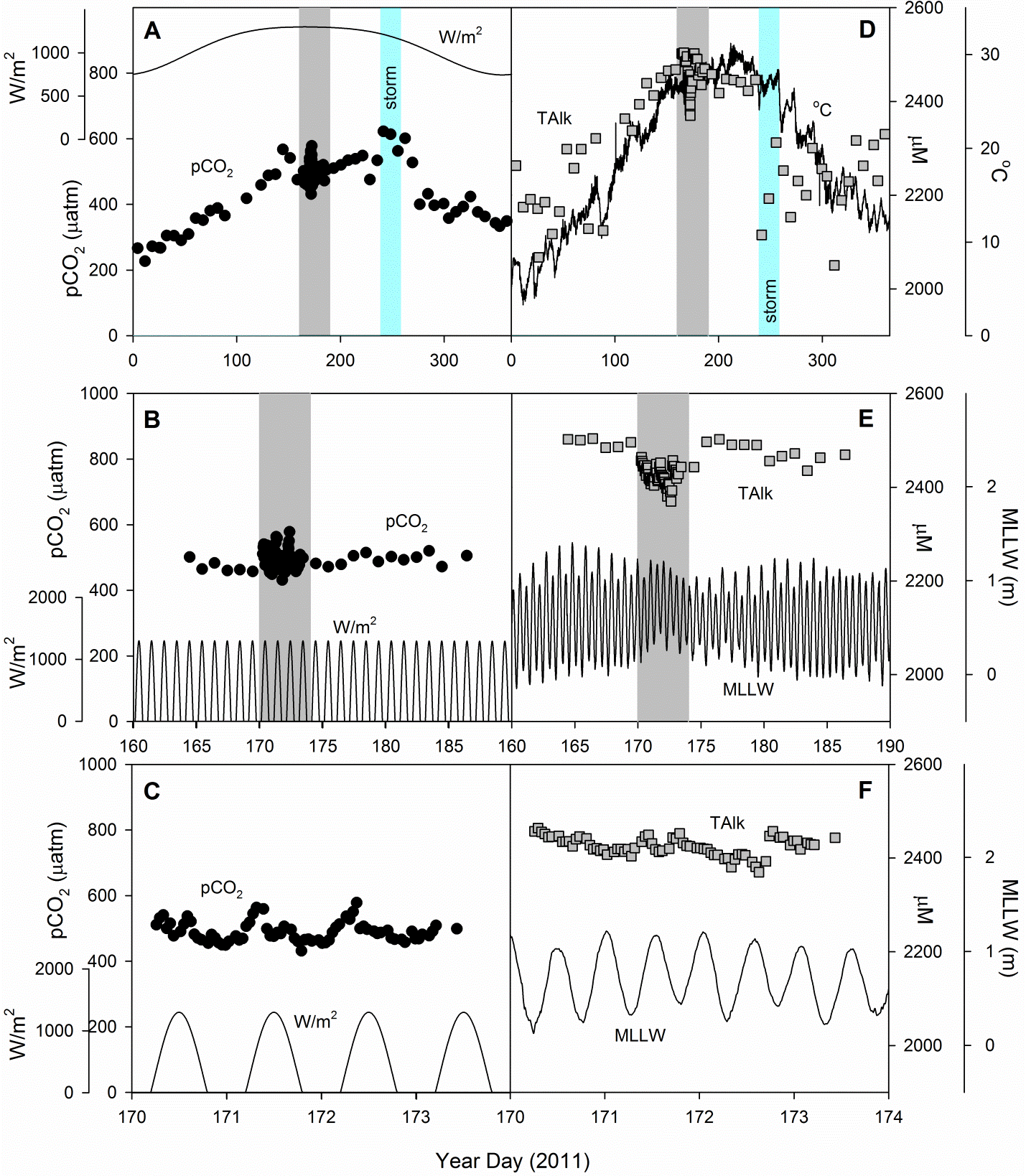

Supplement: Figure S5 — Nested temporal variability of some carbon and environmental parameters at the Pivers Island Coastal Observatory study site for 2011. Nested temporal variability of pCO2 (µatm: A-C), TAlk (µM: D-F) and associated physical variables including incoming no-sky solar radiation (W m-2: A-C), water temperature (°C: D), or tidal height (MLLW, m: E,F). Gray bars indicate periods of more intense sampling. Cyan bars indicate periods influenced by major storm events. Data are shown depicting the nested sampling design with weekly measurements over the course of the year (A, D), daily measurements over a 3 week period (B, E) and hourly measurements over a 3 day period (C, F) Gray bars indicate periods of more intense sampling, shown in the panel immediately below. For clarity, only the maximum daily W m-2 is plotted in top row. (TIF) [file pone.0085117.s005.tif]

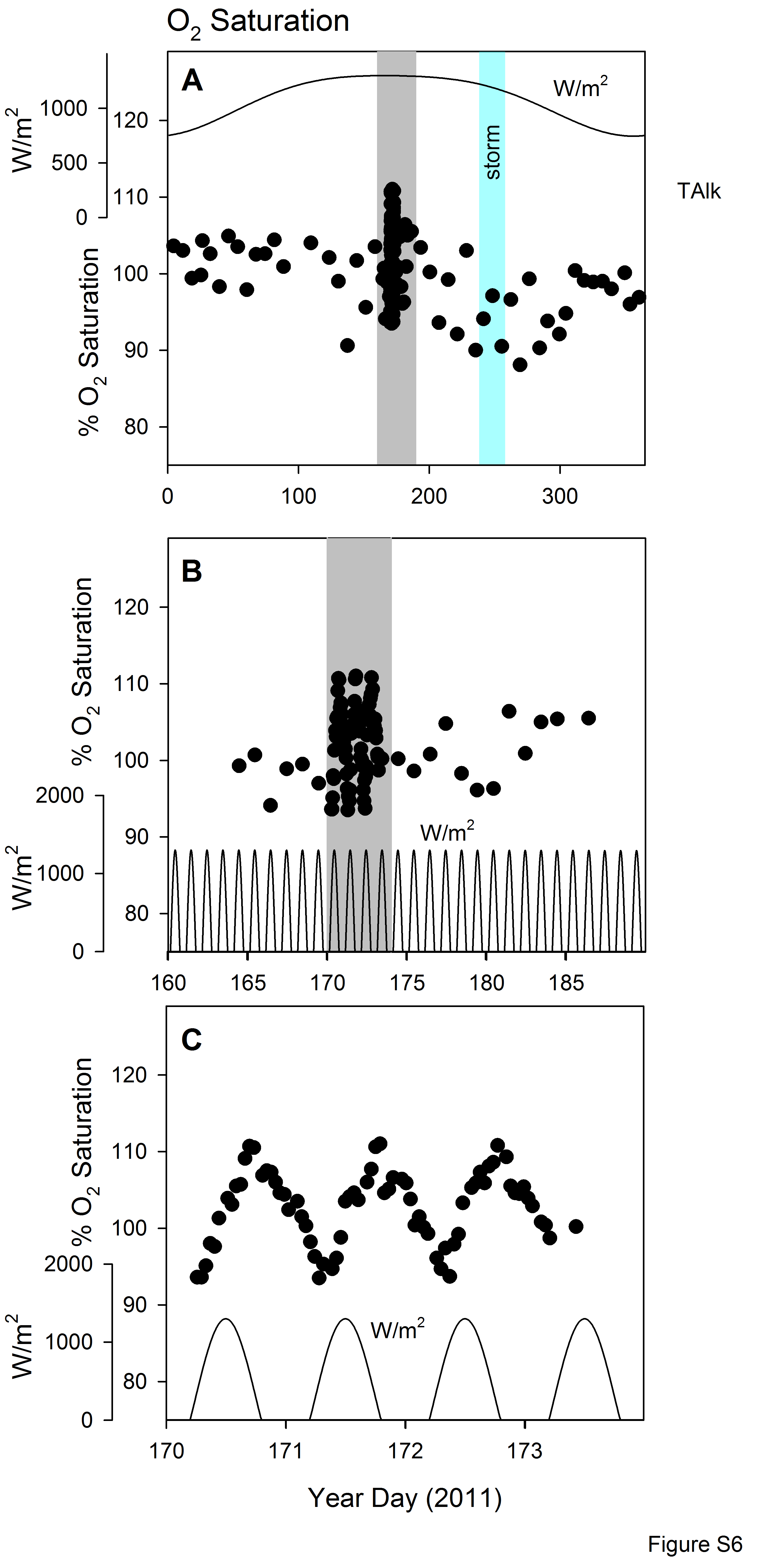

Supplement: Figure S6 — Nested temporal variability of percent oxygen saturation (filled circles) and incoming no-sky solar radiation (W m-2: A-C). Gray bars indicate periods of more intense sampling. Cyan bars indicate periods influenced by major storm events. Data are shown depicting the nested sampling design with weekly measurements over the course of the year (A), daily measurements over a 3 week period (B) and hourly measurements over a 3 day period (C) Gray bars indicate periods of more intense sampling, shown in the panel immediately below. For clarity, only the maximum daily W m-2 is plotted in top row. (TIF) [file pone.0085117.s006.tif]
